# Association between metabolic syndrome and the incidence of gastric cancer: a meta-analysis of cohort studies

**DOI:** 10.1186/s13098-019-0478-y

**Published:** 2019-10-10

**Authors:** Zhibin Li, Hongfeng Han, Yuan Chang

**Affiliations:** 1 Department of Digestive Diseases, The Central Hospital of Luoyang City Affiliated to Zhengzhou University, No. 288 Zhong Zhou Zhong Lu, Luoyang, 471000 China; 2 Department of Dermatology, The Central Hospital of Luoyang City Affiliated to Zhengzhou University, Luoyang, 471000 China

**Keywords:** Metabolic syndrome, Gastric cancer, Cohort study, Meta-analysis

## Abstract

**Background:**

Previous studies investigating the association between metabolic syndrome (MetS) and incidence of gastric cancer (GC) showed inconsistent results. The aim of the study was to evaluate the influence of MetS on GC risk in a meta-analysis.

**Methods:**

Cohort studies that evaluating the association between MetS and GC were identified via systematic search of PubMed, Embase, Web of Science, and Scopus databases. Pooled analyses were performed via a random-effect model or a fixed effect model according to the heterogeneity among the studies. Predefined subgroup analyses were performed to evaluate whether gender or ethnic group of the patients affected the results.

**Results:**

Overall, eight cohort studies with 8,745,671 participants were included, and 37,245 GC cases occurred during follow-up. Results showed that MetS defined by the revised National Cholesterol Education Program’s Adults Treatment Panel III criteria was not associated with a significantly affected GC risk (adjusted risk ratio [RR]: 1.03, p = 0.59; I^2^ = 79%). Subgroup analyses showed that MetS was not associated with a significantly affected risk of GC in male or female patients, and in Asians or Caucasians. Moreover, meta-analysis of four datasets showed that MetS defined by the International Diabetes Federation criteria was also not associated with a significant affected risk of GC (adjusted RR: 0.80, p = 0.05; I^2^ = 0%).

**Conclusions:**

These results indicated that current evidence from epidemiological studies does not support that patients with MetS are at higher risk for the development of GC.

## Introduction

Metabolic syndrome (MetS) refers to a group of metabolic disorders characterized by the manifestations of central obesity, insulin resistance, high blood pressure, and dyslipidemia [1–3]. The prevalence of MetS is continuous increasing globally, which is reported to near 30% in general population [4, 5]. Patients with MetS are vulnerable to various chronic non-infectious diseases, including cardiovascular diseases [6], venous thromboembolism [7], osteoporosis and fractures [8], and cancer [9]. A previous meta-analysis published in 2012 showed that the presence of MetS was associated with increased risks of liver, colorectal, and bladder cancer [9]. Moreover, subsequent analyses showed that the association between MetS and cancer risk may be affected by cancer sites, gender, and ethnic groups of the participants [9]. The meta-analysis failed to show a significant association between MetS and gastric cancer (GC) incidence [9], a common malignancy of the digestive tract [10]. However, only four cohort studies [11–14] were available, including one study that observed the association between MetS and GC mortality [14], which may introduce additional bias and the findings need further validation. In addition, the limited studies prevented further analyses to evaluate whether gender and ethnic groups of the participants may affect the association between MetS and GC. More importantly, a few relevant cohort studies that evaluating the association between MetS and GC since the last meta-analysis [15–19]. Therefore, in the current study, we aimed to evaluate the influence of MetS on GC risk in an updated meta-analysis. With more available datasets, we also explored the potential influences of gender and ethnic groups of the participants on the association of MetS and GC risk.

## Methods

The meta-analysis was designed and performed in accordance with the MOOSE (Meta-analysis of Observational Studies in Epidemiology) [20] and Cochrane’s Handbook [21] guidelines.

### Literature searching

Electronic databases of PubMed, Embase, Web of Science, and Scopus were systematically searched using the combination of the following terms: (1) metabolic syndrome” OR “insulin resistance syndrome” OR “syndrome X”; (2) “cancer” OR “tumor” OR “neoplasm” OR “carcinoma”; and (3) “cohort” OR “prospective” OR “retrospective” OR “follow-up” OR “followed”. We applied this extensive search strategy to avoid missing of potentially related studies. The search was limited to studies published in English. The reference lists of original and review articles were also analyzed manually. The final literature search was performed on August 20, 2019.

### Study selection

Studies were included if they met the following criteria: (1) published as full-length article in English; (2) designed as cohort studies with the minimal follow-up duration of 1 year; (3) included adult participants that were without GC at baseline; (4) participants with MetS were identified as exposure of interest at baseline; (5) participants without MetS at baseline were included as controls; (6) documented the incidence of GC during follow-up; and (7) reported the adjusted risk ratios (RRs, at least adjusted for age and gender) and their corresponding 95% confidence intervals (CIs). Definitions of MetS were consistent with that was applied in the original studies. Reviews, editorials, preclinical studies, and non-cohort studies were excluded.

### Data extracting and quality evaluation

Literature search, data extraction, and study quality assessment were independently performed by two authors according to the predefined inclusion criteria. If inconsistencies occurred, discussion with the corresponding author was suggested to resolve these issues. The following data were extracted: (1) name of the first author, publication year, study location, and study design; (2) characteristics and numbers of the participants, criteria for the diagnosis of MetS, and follow-up period; and (3) number of GC cases during follow-up, and variables adjusted when presenting the RRs. The quality of each study was evaluated using the Newcastle–Ottawa Scale [22]. This scale ranges from 1 to 9 stars and judges the quality of each study regarding three aspects: selection of the study groups; the comparability of the groups; and the ascertainment of the outcome of interest.

### Statistical analyses

The association between MetS and GC incidence was measured by RRs in this study. To stabilize its variance and normalized the distribution, RR data and its corresponding stand error (SE) from each study was logarithmically transformed [21].

The Cochrane’s Q test was performed to evaluate the heterogeneity among the include cohort studies [21, 23], and the I^2^ statistic was also calculated. A significant heterogeneity was considered if I^2^ > 50%. A random effect model was used to pool the results if significant heterogeneity was found; otherwise a fixed effect model was applied. Sensitivity analyses, by omitting one study at a time, were performed to evaluate the potential influence of certain study on the outcome of the meta-analysis [24]. To evaluate the influence of gender and ethnics of the participants on the outcome, subgroup analyses were performed [25]. Potential publication bias was assessed by visual inspection of the symmetry of the funnel plots, complemented with the Egger regression test [26]. The RevMan (Version 5.1; Cochrane Collaboration, Oxford, UK) and STATA software were used for the statistics.

## Results

### Literature search

**Fig. 1:**
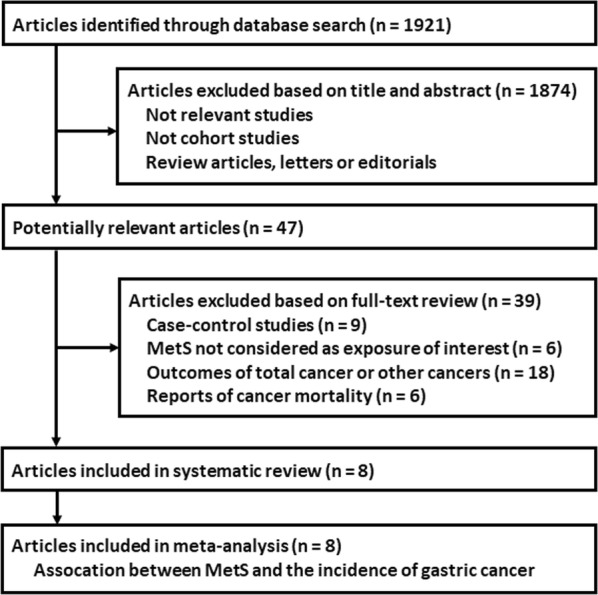
Flowchart of database search and study identification

The flowchart of database search was shown in Fig. 1. Briefly, 1921 studies were obtained from database search, and 1874 of them were excluded due to the irrelevance to the objective of the study. For the remaining 47 potential relevant studies that underwent full text review, 39 were further excluded because nine of them were case–control studies, six did not include MetS as exposure of interest, eighteen reported incidences of total cancer or cancers from other sites, and the other six reported cancer mortality rather than incidence. Finally, eight cohort studies were included [11–13, 15–19].

### Study characteristics and quality

Overall, this meta-analysis included eight cohorts [11–13, 15–19] of 8,745,671 participants, and 37,245 GC cases occurred during follow-up. The characteristics of the included cohorts were shown in Table 1. Four of these cohort studies were performed in Asian countries including Japan [12, 13] and Korea [17, 18], while the other four studies were performed in Europe [11, 15, 16, 19]. All of these studies were of prospective design except for two studies that were retrospective cohorts [13, 17]. All of the studies included general population expect for one study which included patients with vascular diseases [15]. MetS were diagnosed by the criteria of revised National Cholesterol Education Program’s Adults Treatment Panel III (NCEP-ATP III) in all cohorts, while two studies also applied the diagnostic criteria of International Diabetes Federation (IDF) for MetS [12, 13]. Demographic variables such as age and gender were adjusted for all cohorts when presenting the results, most of the studies also adjusted other potential confounding factors such as smoking, alcohol consuming, and family history of cancer [12, 13, 15–19]. The qualities of the cohort studies were generally good, with the NOS ranging from 7 to 9 points.

**Table 1:** Characteristics of the included cohort studies

Study	Country	Design	Characteristics of the participants	Number of participants	Definition of MetS	Follow-up period years	Diagnosis of gastric cancer	Number of gastric cancer cases	Incidence reported	Variables adjusted	NOS
Russo [11]	Italy	PC	Community based population over 40 years	16,677	NCEP-ATP III	1999–2005	Local Cancer Registry	29	M,F,T	Age, gender	7
Inoue [12]	Japan	PC	Community based population	27,724	NCEP-ATP III and IDF	1990–2004	National cancer registries	233	M,F	Age, gender, study area, smoking status, alcohol intake, daily total physical activity level, and TC	9
Osaki [13]	Japan	RC	General health examinees	38,832	NCEP-ATP III and IDF	1992–2007	Tottori prefectural cancer registry	393	M,F	Age, gender, smoking status, and alcohol intake	9
Lindkvist [19]	Norway, Austria and Sweden	PC	Community based population	578,700	NCEP-ATP III	1972–2005	National cancer registries	1210	M,F	Age, gender, study cohort, smoking	8
van Kruijsdijk [15]	the Netherlands	PC	Patients with vascular diseases	6172	NCEP-ATP III	1996–2011	National cancer registries	17	M,F,T	Age, gender, smoking status, and alcohol intake	7
Lin [16]	Norway	PC	Community based population	192,903	NCEP-ATP III	1994–2008	National cancer registries	373	M,F,T	Age, gender, BMI, education, smoking status, and family cancer history	9
Ko [17]	Korea	RC	Community based population	99,565	NCEP-ATP III	2002–2013	Local Cancer Registry	1061	M,F	Age, gender, smoking status, alcohol intake, and exercise	9
Yoo [18]	Korea	PC	National sample cohort for health check-up	7,785,098	NCEP-ATP III	2009–2016	National cancer registries	33,929	T	Age, gender, smoking, alcohol consumption, exercise, family histories (HTN, DM, CVD, cancers), hemoglobin, SCr, TC, LDL-C, and ALT	9

*NOS* the Newcastle–Ottawa Scale, *MetS* metabolic syndrome, *NCEP*-*ATP III* National Cholesterol Education Program’s Adults Treatment Panel III, *IDF* International Diabetes Federation, *PC* prospective cohort, *RC* retrospective cohort, *M* male, *F* female, *T* total, *TC*, total cholesterol, *CVD* cardiovascular diseases, *BMI* body mass index, *HTN* hypertension, *DM* diabetes mellitus, *SCr* serum creatinine, *LDL*-*C* low-density lipoprotein cholesterol, *ALT* alanine aminotransferase

### Association between the revised NCEP-ATP III defined MetS and GC risk

**Fig. 2:**
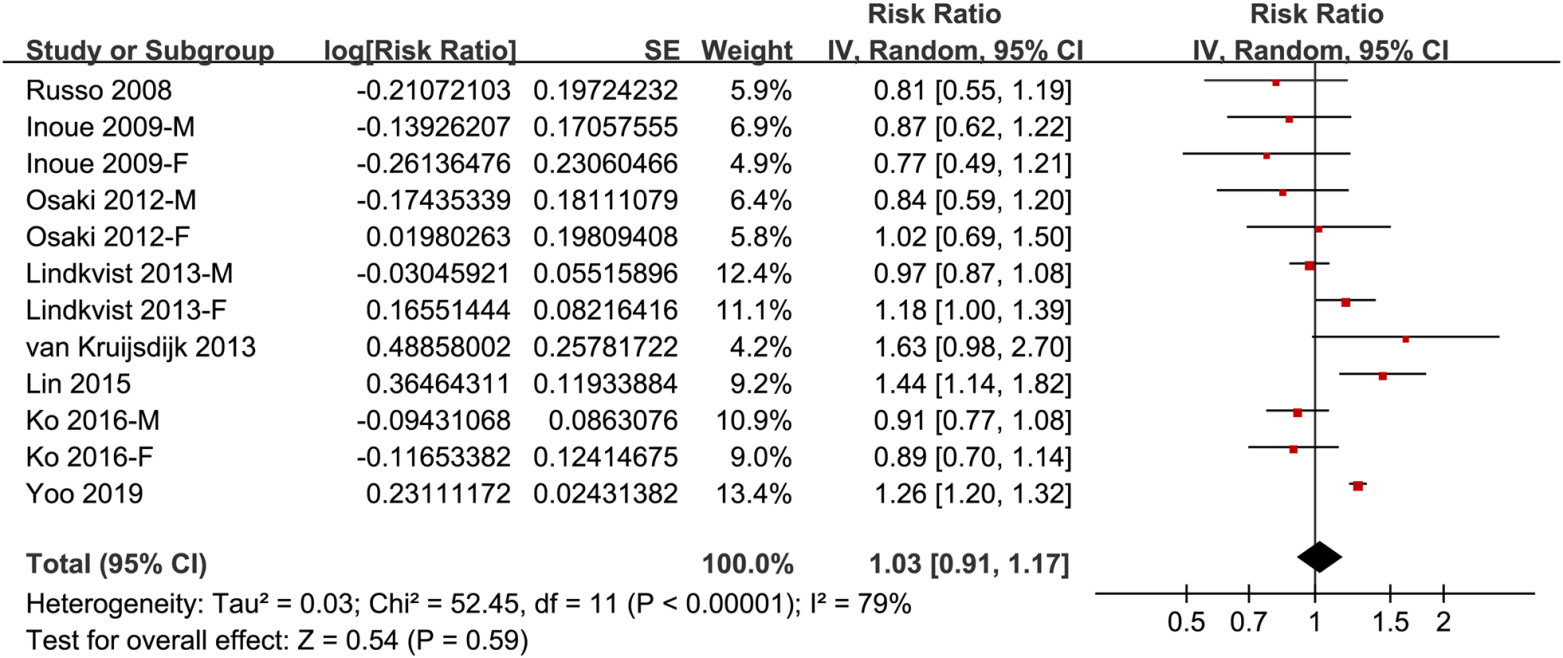
Forest plots for the meta-analysis of the association between MetS defined by the revised NCEP-ATP III and GC incidence in overall population

**Fig. 3:**
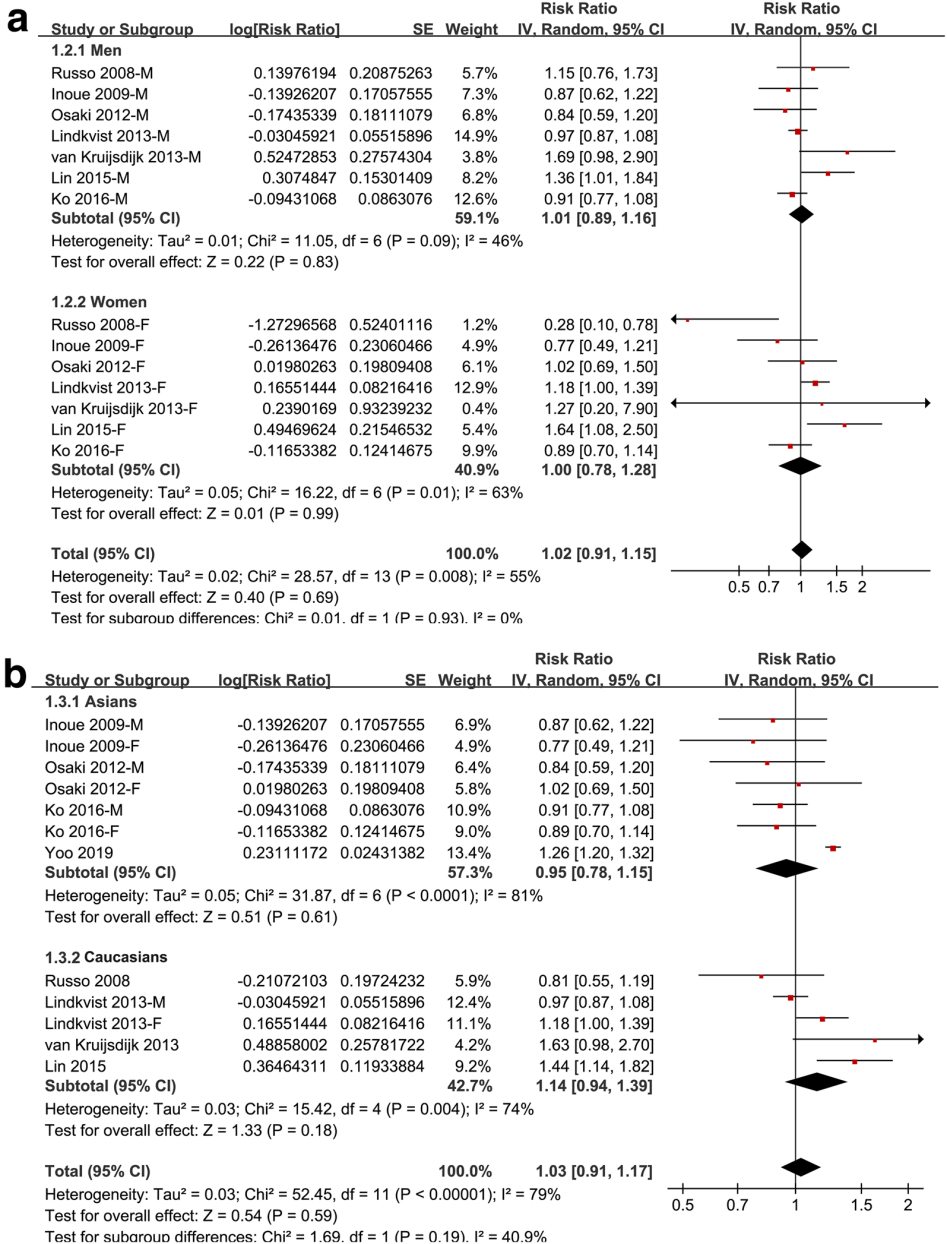
Forest plots for subgroup analyses of the association between MetS defined by the revised NCEP-ATP III and GC incidence. **a** Subgroup analyses by gender; and **b** subgroup analyses by ethnic groups

Meta-analysis of 8 cohorts with 12 datasets showed that MetS defined by the revised NCEP-ATP III was not associated with a significant affected incidence of GC (adjusted RR: 1.03, 95% CI 0.91–1.17, p = 0.59; Fig. 2) with significant heterogeneity (p for Cochrane’s Q test < 0.001, I^2^ = 79%). Sensitivity analyses by excluding one study at a time retrieved similar results (adjusted RR: 1.00–1.05). Results of subgroup analysis according to the gender of the participants showed that Mets was not associated with significantly affected risk of GC in men (adjusted RR: 1.01, 95% CI 0.89–1.16, p = 0.83; I^2^ = 46%) or in women (adjusted RR: 1.00, 95% CI 0.78–1.28, p = 0.99; I^2^ = 63%; Fig. 3a). Similarly, subgroup analysis according to the ethnics of the participants showed that Mets was not associated with significantly affected risk of GC in the Asian participants (adjusted RR: 0.95, 95% CI 0.78–1.15, p = 0.62; I^2^ = 81%) or in the Caucasian participants (adjusted RR: 1.14, 95% CI 0.94–1.39, p = 0.18; I^2^ = 74%; Fig. 3b).

### Association between IDF defined MetS and GC risk

**Fig. 4:**
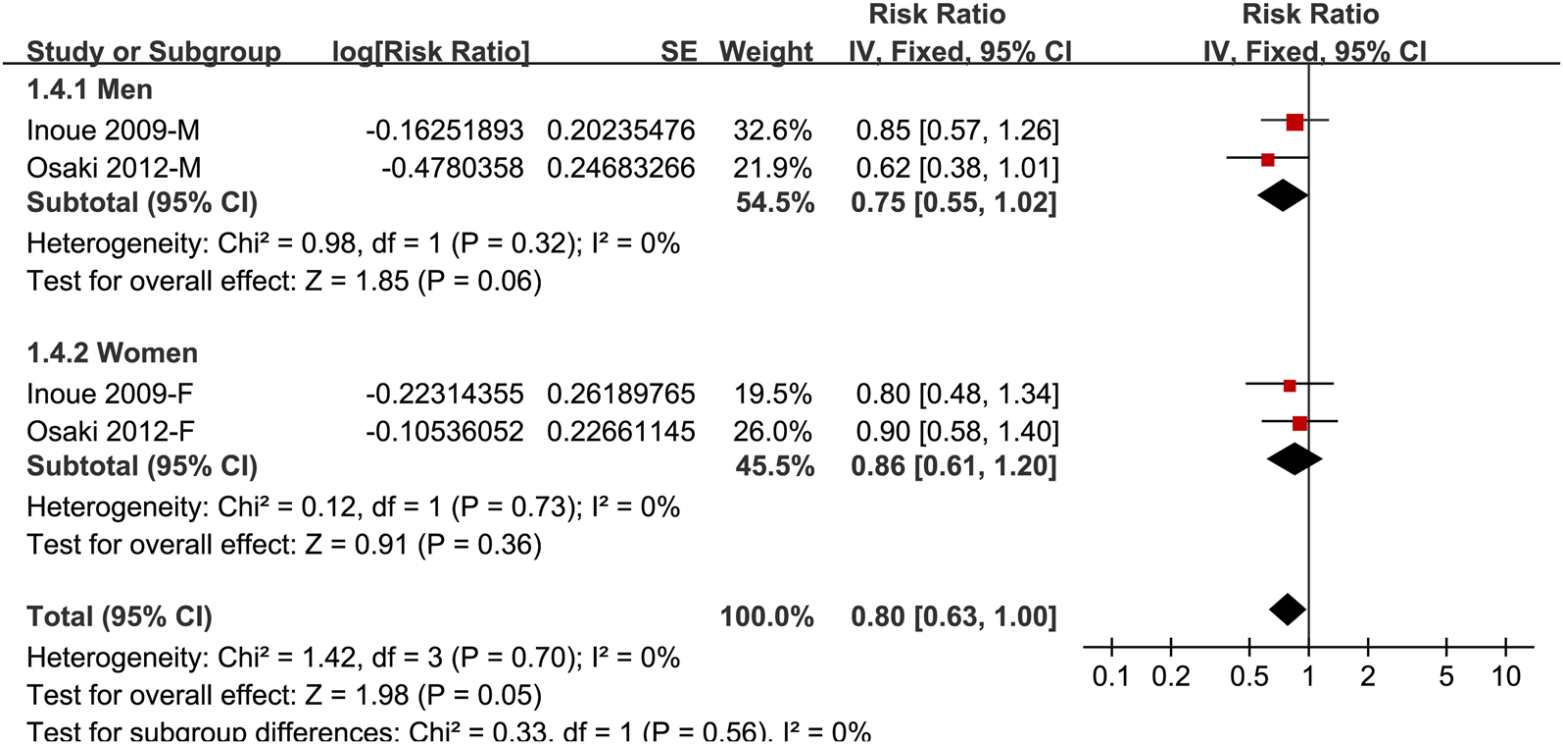
Forest plots for meta-analysis of the association between MetS defined by IDF criteria and GC incidence

The association between IDF defined MetS and GC risk was only reported in two studies [12, 13], which were both performed in Japan. The results of the meta-analysis indicated that IDF defined MetS was also not associated with a significantly affected risk of GC (adjusted RR: 0.80, 95% CI 0.63–1.00, p = 0.05; I^2^ = 0%; Fig. 4), which was not different in men and in women (p for subgroup difference = 0.56; Fig. 4).

### Publication bias

**Fig. 5:**
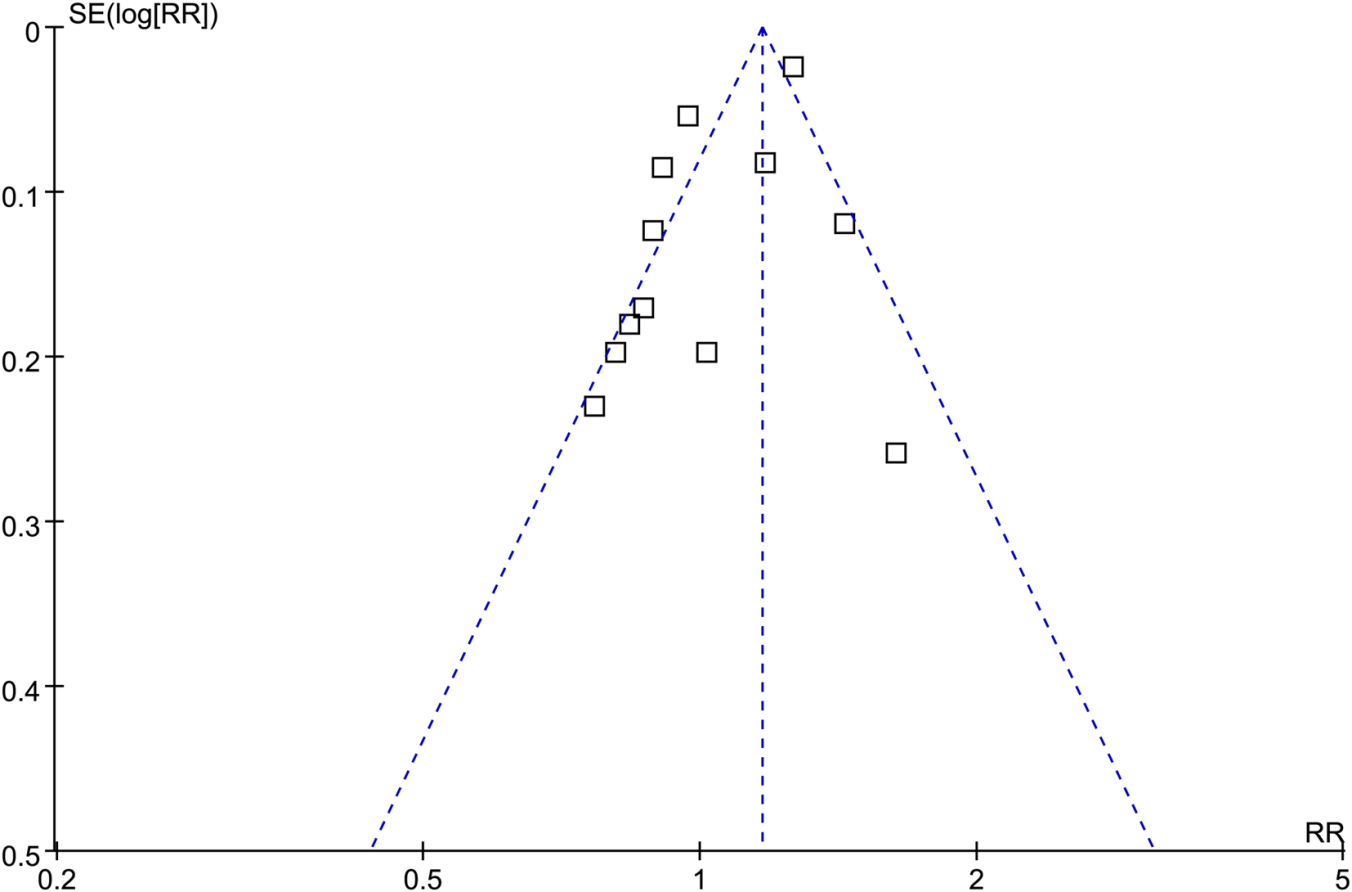
Funnel plots for the meta-analysis of the association between MetS defined by the revised NCEP-ATP III and GC incidence in overall population

The funnel plots for the association between MetS diagnosed by the revised NCEP-ATP III and GC risk were symmetry on visual inspection (Fig. 5), suggesting insignificant publication bias. Results of Egger’s regression test showed similar results (p = 0.369). Publication bias for the meta-analysis of IDF defined MetS and GC risk was difficult to estimate since only four datasets were included.

## Discussion

In this study, by including all relevant cohort studies, our meta-analysis showed that MetS defined by the revised NCEP-ATP III criteria was not associated with an increased risk of GC. The robustness of the finding was indicated by results of sensitivity analyses, and subgroup analyses according to the gender and ethnic groups of the participants. Result of meta-analysis also showed that IDF defined MetS was not associated with a significantly affected risk of GC. Taken together, these results showed that MetS is not a risk factor of GC in general population.

To the best of our knowledge, our study is the first comprehensive meta-analysis that focused on the association between Mets and incidence of GC. Our study has the following strengths. Firstly, we only included cohort studies reporting the GC incidence other than GC mortality. Since the mortality of GC was determined by complex factors including therapeutic status, excluding studies reporting GC mortality avoided introducing of additional bias. Secondly, the inconsistent findings of previous studies regarding the association between MetS and GC risk may be explained by the variability of sample sizes of the studies. An insignificant finding may be obtained if the study was statistically underpowered. Our meta-analysis included twelve datasets with over eight million participants, which minimized the chance that the unaffected GC risk in MetS participants was caused by inadequacy of statistical power. Thirdly, we combined RR data that was adjusted most adequately to minimize the confounding factors for the association between MetS and GC risk. Finally, we performed meta-analysis according to the definitions of MetS, and subgroup analyses according to the gender and ethnic groups of the participants. None of these analyses demonstrated an increased risk of GC in MetS participants, suggesting that the findings were reliable. The insignificant association between MetS and GC risk may be a reflection of inconsistent association between individual components of MetS and GC incidence. Although obesity has been shown to be a risk factor for GC incidence [27, 28], diabetes or insulin resistance was shown to have no significant influence on GC incidence [29]. As for the relationships of blood pressure, serum triglyceride, or high-density lipoprotein cholesterol with GC incidence, rare studies were performed and mostly did not show any significant findings [30]. A recently published meta-analysis evaluated the influence of MetS on survival in patients with digestive tract cancer [31]. Although the results showed that MetS was associated with increased cancer-specific mortality in overall patients, MetS was not associated with an increased risk of mortality in GC patients [31]. Taken together, current evidence from epidemiological studies did not support a significant association between MetS and an increased risk of GC.

Our meta-analysis has limitations. Firstly, as a nature of meta-analysis of observational studies, we could not exclude other factors that may confound the association between MetS and GC risk, such as treatments with metformin. Secondly, although we analyzed MetS by revised NCEP-ATP III or IDF criteria separately, association between MetS defined by other criteria and GC risk should also be explore. Thirdly, only four datasets from two studies were included for the association between IDF defined MetS and GC risk. Therefore, these findings should be confirmed in future studies. Fourthly, GC is a heterogeneous disease. The association between MetS and different subtypes of GC should be evaluated in the future. Finally, some commonly known risk factors for GC (including *Helicobacter pylori* infections, gastric ulcers, or eating disorders) were not well controlled in the included studies. Well-designed large-scale cohort studies with adequate adjustment of these factors should be performed to validate our findings.

## Conclusions

In conclusion, results of our meta-analysis showed that presence of MetS is not associated with an increased risk of GC in general population. The influences of each component of MetS on pathogenesis of GC should be evaluated in future studies.

## Data Availability

The available data and materials section refers to the raw data used in our study are included in manuscript with tables, figures and its supplementary information files. All the authors agreed that the data could be shared if researchers required.

## Abbreviations

MetS: metabolic syndrome
GC: gastric cancer
MOOSE: Meta-analysis of Observational Studies in Epidemiology
RRs: risk ratios
SE: stand error
NCEP-ATP III: revised National Cholesterol Education Program’s Adults Treatment Panel III
IDF: International Diabetes Federation
